# Modification of asphalt mixtures for cold regions using microencapsulated phase change materials

**DOI:** 10.1038/s41598-019-56808-x

**Published:** 2019-12-30

**Authors:** Moises Bueno, Muhammad Rafiq Kakar, Zakariaa Refaa, Jörg Worlitschek, Anastasia Stamatiou, Manfred N. Partl

**Affiliations:** 1 Empa, Swiss Federal Laboratories for Material Science and Technology, CH-8600 Dübendorf, Switzerland; 2 Chalmers University of Technology, Gothenburg, SE-412 96 Sweden; 3 Lucerne University of Applied Sciences and Arts, CH-6048 Horw, Switzerland

**Keywords:** Chemistry, Energy science and technology, Engineering, Materials science, Physics

## Abstract

Phase change materials (PCMs) may be used to regulate the temperature of road surfaces to avoid low-temperature damages when asphalt materials become brittle and prone to cracking. With this in mind, different asphalt mixtures were modified with microencapsulated phase change materials (i.e. tetradecane) to assess their thermal benefits during the phase change process. Likewise, the effect on the mechanical performance of PCMs as a replacement of mineral filler was assessed. Special attention was paid to dry and wet modification processes for incorporating the PCMs into the mixtures. The results showed that PCM modifications are indeed able to slow down cooling and affect temperatures below zero. Approximately, a maximum of 2.5 °C offset was achieved under the tested cooling conditions compared to the unmodified reference specimens. Regarding the mechanical response at 0 °C and 10 °C, the results indicated that the PCM modification significantly reduces the stiffness of the material in comparison with the values obtained for the reference mixture.

## Introduction

Asphalt road surfaces are directly exposed to environmental conditions. The ambient temperature has a great influence on the physicochemical properties of these materials. Asphalt binders (*i.e*. bitumen) are temperature-sensitive materials that show viscous properties at high temperatures and become elastic in nature during low temperatures. In winter, the asphalt becomes stiff and brittle, eventually resulting in thermal cracking and traffic-related distresses such as fatigue damage. Moreover, during cold seasons, the formation of ice on the road surface may reduce the friction at the tire-surface interface, which considerably compromises traffic safety.

Phase change materials (PCMs) are latent heat storage materials that absorb or release heat under almost isothermal conditions. In terms of adjusting or regulating temperature, PCMs show great potential to avoid or delay the extreme temperature occurrence. They have a narrow characteristic temperature range where melting and crystallization occur. This temperature change can be selected to regulate operating temperatures of different applications by choosing a particular PCM. Pielichowska and Pielichowski^1^ summarized a number of thermal, physical, kinetic and chemical criteria that a PCM should meet for being used as thermal energy storage material. PCMs have been widely used in a variety of applications such as textile/fabrics, computer appliances, solar energy, pharmaceutical industry or aerospace^2^. For construction materials, microencapsulated PCMs have been incorporated in building elements such as panels, plasterboards and wallboards^3–6^. Moreover, the experimental results of new designs of capsules with PCMs have revealed their potential for thermal energy storage (TES) systems^7–9^.

In asphalt roads, several numerical models have been developed to predict temperatures under real weather conditions that take into account the presence of PCMs in asphalt concrete pavements^10,11^. However, although the benefits of PCMs for modifying asphalt roads appear very promising and would prevent temperature-related failures, their practical feasibility in this field has been addressed only recently. At low temperatures, an alternative idea of using PCM-modified concrete was proposed by Bentz and Turpin^12^ to reduce the number of freeze/thaw cycles in concrete bridge decks. Using numerical simulations, they showed that 15 wt% microencapsulated PCM-modified concrete could potentially reduce the number of freeze/thaw cycles by up to 30% compared to plain concrete (control). Focused on asphalt materials, Chen *et al*.^13^ found that the use of different organic acids (T_melt_ = 45 and 50 °C, H_melt_ = 110 and 100 J/g, respectively) as PCM was promising in solving problems of road surfaces at high temperatures. Their results showed that different PCMs had diverse effects on the thermal performance of asphalt mixtures. Besides this, the addition of PCMs to asphalt mixtures resulted in a decreased indirect tensile strength, a weakened rutting resistance, and in a reduced cracking resistance at low temperatures. Practical issues related to the incorporation of PCMs in the preparation process for road constructions were first evaluated by Ma *et al*.^14^. They carried out different studies, focusing on the conception of feasible procedures to add PCMs to asphalt mixtures. Different composites were developed by using unsaturated organic acid (details not available) as PCM impregnated in a polypropylene carrier. However, they found that part of the polypropylene can melt at high temperatures affecting the mechanical properties of the asphalt mixture. In a later study^15^, they incorporated tetradecane (T_melt_ = 5.8 °C and H_melt_ = 221.2 J/g) as PCM in porous materials such as silica and activated carbon in order to obtain a shape stabilized PCM. Furthermore, they covered the PCM composite using an ethylcellulose membrane with a melting temperature exceeding 160 °C, preventing the breakage of the composite during the mixing procedure. Likewise, Kakar *et al*.^16–18^ recently investigated the use of tetradecane as PCM in asphalt mixtures for low-temperature applications. They also studied the incorporation process, the type of binder as well as the aging effects. It was found that the direct interaction of tetradecane with asphalt binder not only significantly affected the rheological properties of asphalt binders but also did not provide the expected thermal energy storage in a latent form. Therefore, to prevent possible leakage due to breakage of the capsule shell, the use of microencapsulation of such PCMs was found crucial for asphalt binder modification.

The latest works on the addition of PCMs as thermal modifier in asphalt materials are focused on the use of polyethylene glycol (PEG) supported by different mineral carriers^19–21^. In these new studies, the obtained composite PCM replaces some of the original fine aggregates. It has been reported that this approach improved the thermal properties of the modified mixes. Nevertheless, the volume fraction of the fine aggregates in the road materials is limited (approximately 9% by mass of overall aggregates) and limits the volume fraction of the PCMs. In another study by Li *et al*.^22^, reinforced PCM microcapsules were developed to replace a part of the coarse aggregates, obtaining modified mixtures that satisfied the technical requirements.

The current work aims to experimentally evaluate the influence of microencapsulated tetradecane as PCM on the rheological, mechanical, and thermal performances of asphalt materials for road surfaces at low temperatures. The approach involves the total replacement of the mineral filler (particles with sizes below 0.063 mm) with microencapsulated PCM as artificial filler. First, different modified asphalt mastics (asphalt binder plus filler) were prepared in order to conduct a series of rheological measurements. Afterwards, thermal and mechanical properties of asphalt mixtures modified with low temperature PCM were analysed. Moreover, to assess the survival of the microcapsules that underwent the mixing stage, different incorporation processes (dry and wet methods) were used for the preparation of the asphalt mixtures. By modifying the asphalt mixtures with PCMs, it is expected that, on the one hand, the mechanical performance at low temperature can be improved as a direct consequence of a) the use of flexible microcapsules as artificial filler and b) the temperature control which, in parallel, would prevent thermal cracking effects during cooling. Furthermore, the modification will delay (or completely avoid) the onset of temperatures below zero, i.e. the formation of ice on the road surface. This would reduce the safety risks and economic losses^23–25^ as well as the use of salts or chemical compounds such as anti-freezing agents that may contaminate soils and groundwater^26^.

## Results and Discussion

**Figure 1:**
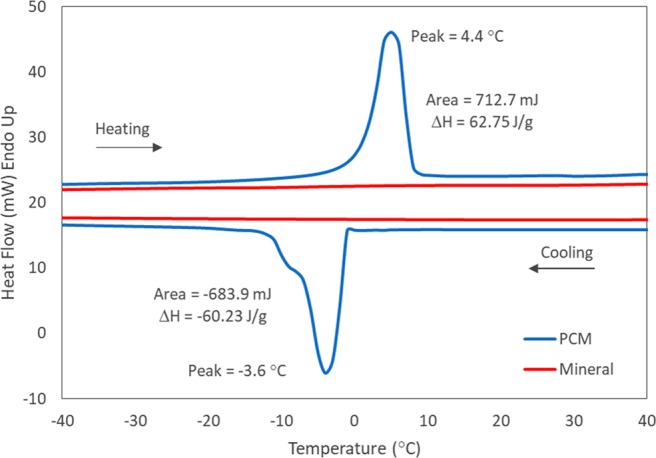
Heat flow versus temperature for mineral (reference) and PCM asphalt mastics.

The thermal analysis techniques enable the characterization and quantification of the thermal transition of the different mastic test samples. The samples were analysed using differential scanning calorimetry (DSC) and heat flow was recorded during the heating and cooling proceses. In Fig. 1, the heat flow versus temperature is given for both mineral filler and PCM asphalt mastics. Whereas the conventional mastic with mineral filler does not show any thermal transition, upon cooling, the PCM mastic shows an exothermic peak below 0 °C, due to the crystallization of tetradecane. The integration of the peak area resulted in a crystallization enthalpy of 60.2 J/g at a peak temperature of −3.6 °C. The parameters are directly related to the thermal energy stored by the PCM, and in the modified asphalt mastic, which will depend on the content of the PCM as well as on its melting enthalpy. During heating, the observed endothermic enthalpy was 62.7 J/g at a peak temperature of 4.4 °C. The temperature difference between the melting temperature and crystallization is called supercooling, and can be reduced by decreasing the cooling and heating rate as well as by using nucleating agents. It was found that the observed melting enthalpy of 62.7 J/g for PCM/binder ratio of 0.5 was in agreement with the expected melting enthalpy of 39.1 J/g observed for PCM/binder ratio of 0.25^18^.

**Figure 2:**
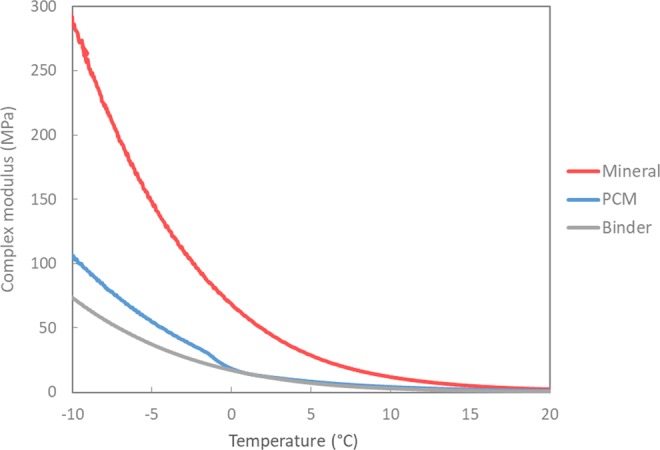
Low-temperature sweep of binder, mastics with mineral (reference) and PCM.

The rheological measurements using a dynamic shear rheometer (DSR) temperature sweep test are illustrated in Fig. 2. The results infer that upon cooling, the complex modulus (G*) of the unmodified asphalt binder increases with the decrease in temperature. Clearly visible is the stiffening effect of the mineral filler on the performance of the conventional mastic with higher values of G* when compared to the asphalt binder sample (without any filler). Also, it can be observed that the PCM modified asphalt mastic shows a similar response to that of the unmodified binder at high test temperatures (10 °C to 0 °C). However, upon further cooling, the stiffness of PCM modified mastic increases and remains in-between the unmodified binder and the mineral filler modified mastic. According to these results, the crystallization effect of the PCM can be observed through the change of G* in the temperatures from −1 °C to −3 °C (cf. Fig. 2). This result is consistent with the crystallization temperature (peak = −3.6 °C) observed for the PCM mastic in the previous thermal analysis (cf. Fig. 1). This difference in temperatures at which the phase change occurs could be related to the lower cooling rate used here compared to the DSC analysis. In any case, it is hypothesized that this stiffening effect is associated with the phase change of microencapsulated PCM, from liquid to solid (i.e. crystallization of tetradecane). This phenomenon also involves a release of the thermal energy stored which will lead to an increase in the overall sample temperature. In this sense, it is significant to remark that the temperature shown in Fig. 2 does not correspond to the actual temperature of the test sample, but rather to the temperature of the lower plate of the setup.

**Figure 3:**
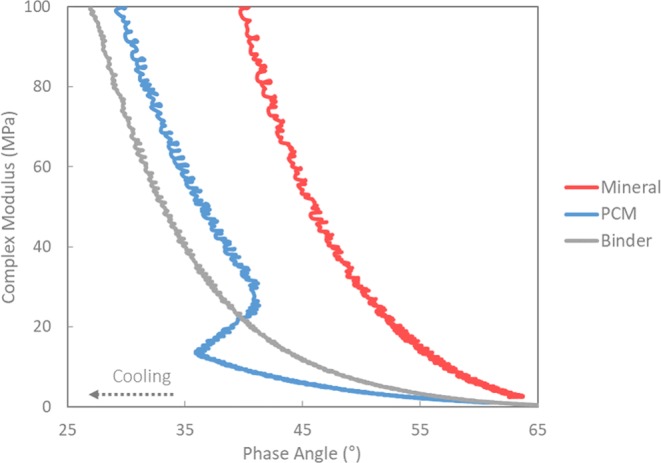
Black diagram of binder, mastics with mineral (reference) and PCM.

In order to see the effect of PCM crystallization more clearly, the Black diagram presented in Fig. 3 provides an appropriate means for comparing the rheological properties of modified binders (mastics) without requiring mathematical shifting, like, e.g., in case of modulus-frequency master curves. On the one hand, it can be seen that the presence of flexible microcapsules (liquid PCM) within the asphalt matrix of the PCM mastic causes a lower stiffness even compared to the asphalt binder sample (without filler). On the other hand, the thermal effect of PCM is more significant in this type of plot, where the temperature range of PCM crystallization becomes evident through a sudden increase of the phase angle due to the increase in temperature (thermal energy release) as well as the parallel and abrupt increase in G* (solid PCM). Therefore, the delayed change in phase angle with respect to the G* indicates the impact of the microencapsulated PCM crystallization within the asphalt matrix.

**Figure 4:**
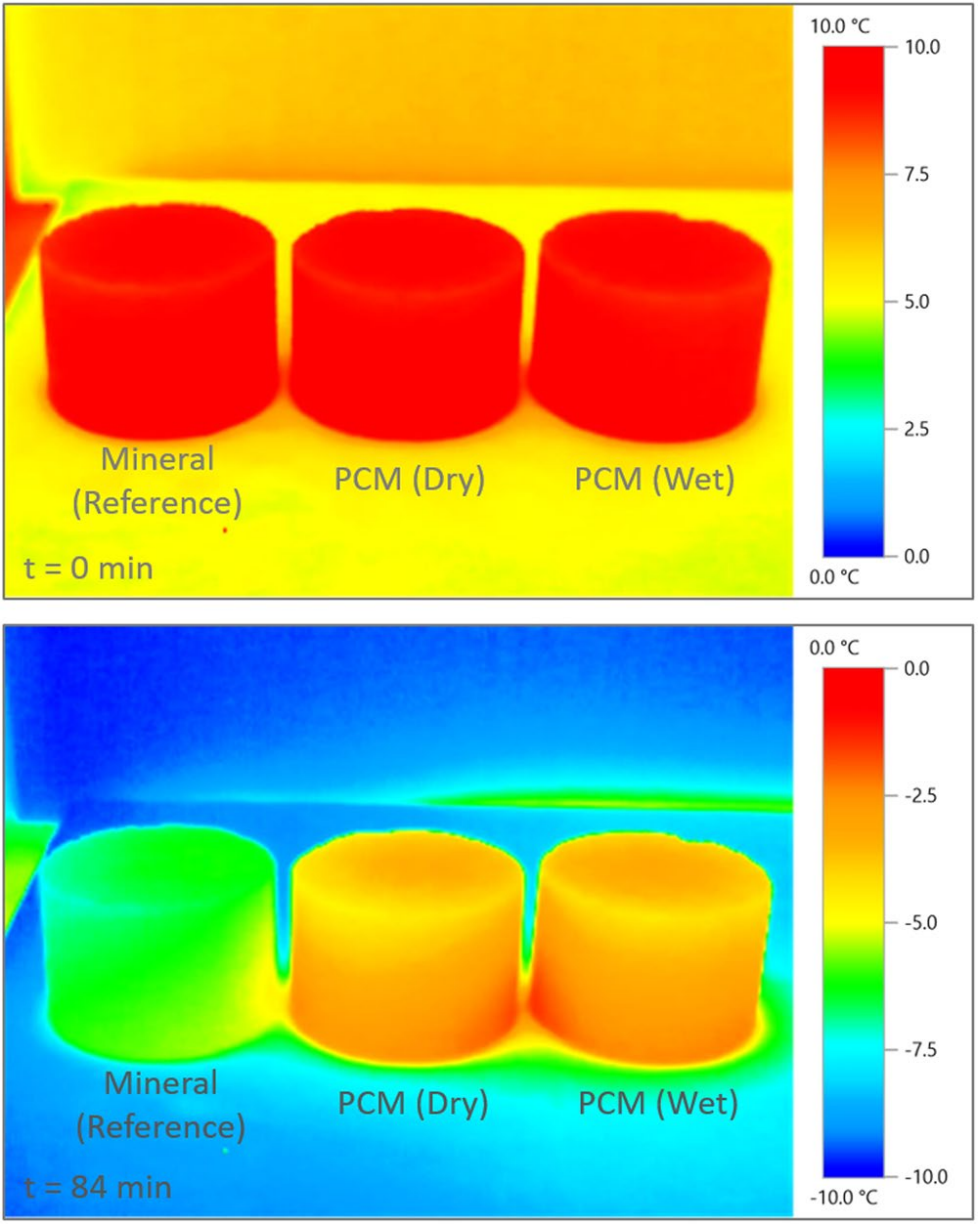
Thermal Infrared images of mixtures with mineral (reference) and PCM (dry and wet).

Regarding the thermal response of the PCM modified asphalt mixtures upon cooling, two IR images corresponding to different moments during the thermal cycle are shown in Fig. 4. In this study, from the reference mixture with mineral filler, only specimens prepared by dry method (conventional process) were evaluated. It can be seen clearly that all the specimens were at the same temperature (10 °C) after the conditioning time at the beginning of the cooling ramp (t = 0 min). Nevertheless, after 84 min of cooling, which involves an air temperature close to −10 °C, it is visibly evidenced that PCM modified specimens have a surface temperature difference of more than 2 °C. These images confirm the efficiency of the PCM as thermal a modification of the asphalt mixture by delaying the temperature decrease. As it was observed in the analysis of the rheological measurements, the release of thermal energy during the phase change of the tetradecane allowed the specimens to keep their temperature despite the continuing decrease of the surrounding air temperature.

**Figure 5:**
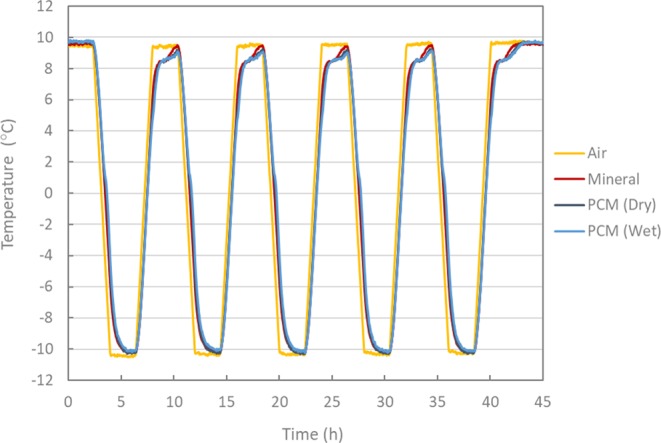
Thermal cycles of mixtures with mineral (reference) and PCM (dry and wet).

**Figure 6:**
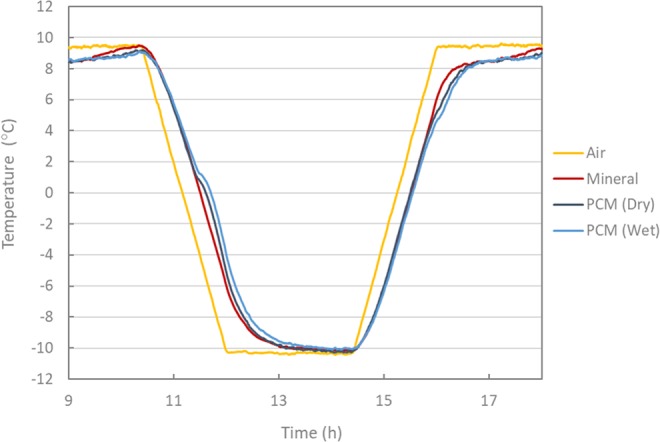
Second thermal cycle of mixtures with mineral (reference) and PCM (dry and wet).

**Figure 7:**
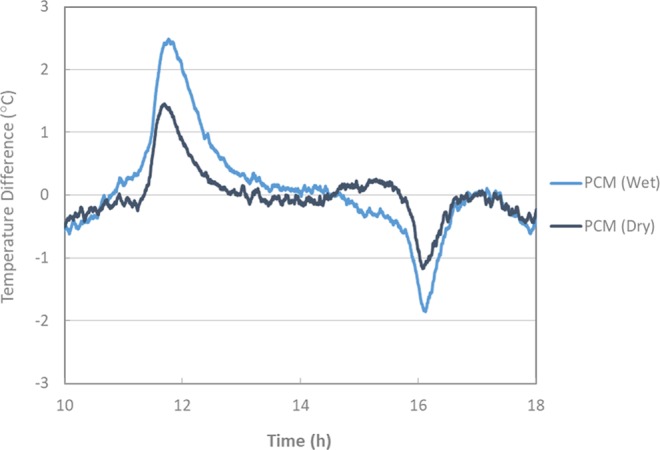
Temperature difference evolution between mixtures of PCM (dry and wet) with mineral (reference).

In order to analyse the previously observed effect in detail, the evolution of the surface temperatures associated with the different test samples during the conducted thermal cycles is shown in Fig. 5. During the cooling phase between 10 °C to 1 °C, no clear difference can be found between the curves of mineral (reference) and PCM modified asphalt mixtures. However, at around 1 °C, the slope of the curves of PCM modified mixtures starts to deviate from the mineral (reference) mixture and rejoins it back after approx. 90 min at −10 °C. This can be better observed in Fig. 6 where only one complete thermal cycle is shown. The phase change process seems to start at a higher temperature than in the previous experiments (DSC and DSR). This observation may be explained by the fact that the actual crystallization temperature (starting at −1 °C) is influenced by the cooling rate. On the other hand, during heating, the effect of phase change is observed at around 5 °C and continued until the mixture temperature reaches 10 °C. This is because the PCM modified mixtures exhibit a relatively low-temperature change rate during cooling and heating. This phenomenon is attributed to the effect of PCM crystallization and melting. Upon cooling, the PCM releases energy in the form of heat that was stored during the melting process. Taken as benchmark a temperature of 0 °C (i.e. ice-formation), a delay of approximately 15 min was obtained by the mixtures modified with PCM (wet process) compared to the reference mixtures. Apparent deviations can be observed between the reference and PCM modified mixtures when the actual temperature differences between them are plotted, as they are in Fig. 7. These temperature differences remained higher for the mixtures modified with PCM (wet process) than for those prepared using the dry process. Deviations up to 2.5 °C were recorded by the PCM asphalt mixtures modified by the wet process. This can be attributed to the fact that the wet process of PCM modification has a lower chance of microcapsule breakage during the mixing process. Moreover, in the dry process, the microencapsulated PCMs are added directly to the mineral aggregates during the mixing, thus making them more vulnerable to breakage due to the squeezing of aggregates. This fact would lead to a reduction of the effective PCM concentration resulting in a lower thermal effect^16^.

**Figure 8:**
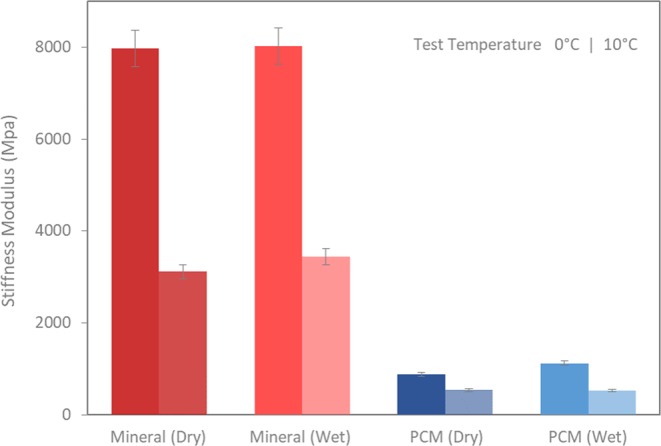
Indirect tensile stiffness moduli of mineral (reference) and PCM mixtures (dry and wet).

The IT-CY stiffness modulus is used as an important index for evaluating low temperature cracking susceptibility of asphalt mixtures^27^. Cyclic indirect tensile stiffness was applied for both conventional and modified mixtures as described later at 10 °C and 0 °C in order to cover the expected phase change of the tetradecane when PCM was in liquid and solid-state respectively. Figure 8 represents the IT-CY stiffness modulus results of asphalt mixtures with PCM modification and the mineral (reference) using dry and wet processes. It is worth noting that, for comparison, a set of reference specimens (Mineral-Wet) were prepared while adding the required amount of filler to the binder first and then mixing with aggregates. The results indicate that the stiffness moduli of the mineral (reference) mixtures prepared using both dry and wet procedures are similar. This confirms that the incorporation process of the mineral filler does not play a major role in conventional mixtures.

Nevertheless, at test temperature 0 °C, slight variations were observed between the PCM modified mixtures prepared by wet and dry processes. It seems that the PCM modified mixtures (dry process), when compared to the wet process, have slightly lower values of stiffness moduli at 0 °C. On the one hand, the breakage of the microcapsules during the dry process would promote the softening of binder due to the leaked PCM and could result in lower stiffness compared with those prepared by the wet process. In parallel, the lower presence of the microcapsules would also involve a lower concentration of PCM in the solid-state after crystallization compared with the PCM modified mixtures prepared by the wet process. On the other hand, the comparable stiffness results at 10 °C suggest that there could be a balance between the effects of the flexible microcapsules (PCM liquid) and the softening of binder due to leakage of the microcapsules.

Overall, it was found that the PCM modified asphalt mixtures resulted in significantly lower values of stiffness compared with conventional mineral (reference) mixtures. This significant reduction in moduli must be attributed to the presence of microencapsulated PCM in the modified mixtures. The rheological response of the PCM modified asphalt mastic (cf. Fig. 3) already anticipated the mechanical performance observed in the modified mixtures. Those results suggested that the use of PCM as filler leads to lower complex shear modulus at low temperature compared to the mineral asphalt mastic with high stiffness properties, which is positive in terms of cracking susceptibility. Furthermore, since the PCM is added based on the equivalent volume replacement of fillers, the resultant modified mixtures will present different volumetric properties. In this regard, Chen *et al*.^13^ in their research, reported that the addition of PCM to asphalt mixtures also resulted in a decreased indirect tensile strength and a weakened rutting resistance.

In conventional asphalt mixtures, the mineral filler plays a determinant role that directly affects their final mechanical properties^28,29^. They are mainly needed in order to fill the voids in between the coarser aggregates, stabilise the mixture as well as reduce the optimum binder content. Moreover, it is commonly known that they tend to stiffen the asphalt mixture by means of an improvement of the strength within the asphalt-aggregate system. In the current work, it was observed that the total replacement of mineral filler with microencapsulated PCM significantly reduced the stiffness at low temperature which directly influences mechanical performance. Based on the performance aspect, the low stiffness response in the low-temperature regime is an expected behaviour.

Nevertheless, at high temperatures, this property for asphalt materials needs to be high enough in order to avoid severe rutting or permanent deformation. In this sense, since the presence of mineral filler seems to be required to avoid an undesirable performance at high temperatures due to the expected lower stiffness, it would be recommended to use PCM as a partial replacement or as an additive in asphalt mixtures. Furthermore, as the amount of mineral filler in an asphalt mixture (usually between 6–9%wt. of mix) ultimately limits the amount of PCM to be added, the use of lightweight aggregates as PCM carrier material could be an interesting option that not only could replace coarse mineral aggregates but also would allow the use of large amounts of PCM.

## Conclusions and Recommendations

The use of phase change materials (PCMs) in asphalt pavements has shown considerable potential for future applications. The current study explored the thermal performance of microencapsulated PCM modified asphalt mastics and dense-graded mixtures as well as their mechanical performance in indirect tension tests. The effect of microencapsulated tetradecane (low-temperature PCM) as a replacement of mineral filler was assessed, and its incorporation process (wet and dry methods), was experimentally analysed. The differential scanning calorimetry (DSC) results confirmed the thermal influence of the PCM on the modified mastics during the phase change process (melting and crystallization). Moreover, the analysis of the rheological responses revealed that the thermal energy released during the phase change affected the viscoelastic properties of the samples. It was also observed that the complex modulus of PCM mastic increases after the crystallization. Furthermore, the surface temperature measurements of the PCM modified asphalt mixtures using the wet process evidenced a thermal difference of more than 2 °C from the reference mixture and a time delay in reaching temperatures below zero. This effect was found to be less pronounced when the incorporation of the PCM was carried out using the dry process. Moreover, this effect remained unchanged after five thermal cycles. Finally, a drastic reduction of the stiffness modulus at low temperature was observed as a consequence of the total volumetric replacement of the mineral filler with microencapsulated PCM. In this sense, achiving the mechanical performance of this type of PCM modified mixtures at moderate or high temperatures might be challenging. Therefore, although the thermal effect was found promising, it is recommended to incorporate microencapsulated PCM only as an additive to ensure the presence and function of the mineral filler within the asphalt mixtures. Additionally, in order to increase the amount of PCM, the choice of porous coarse aggregates as a PCM carrier could be an interesting approach to be investigated in the future. In that case, the challenge would be to find aggregates that allow the absorption of PCM without jeopardizing the strength of the asphalt material.

## Materials and Methods

**Figure 9:**
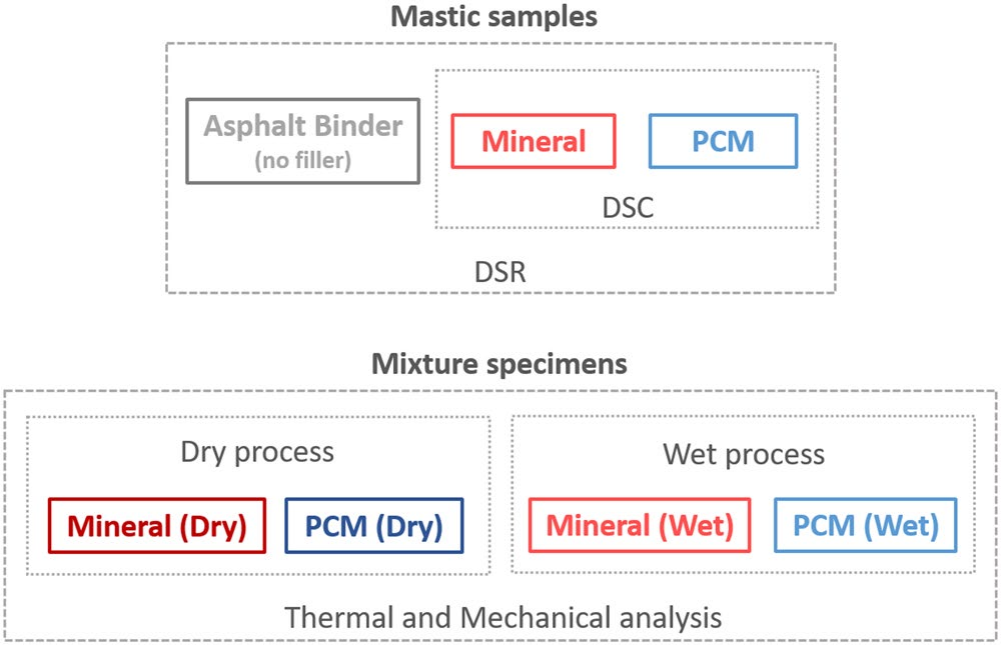
Summary of the research plan conducted in this study.

An overview of the research plan including the different asphalt mastics and mixtures prepared for this work, along with the corresponding experimental analysis carried out is shown in Fig. 9. Further details are explained in the following sections.

### Mastic and mixture preparation procedures

In this study, Microtek Laboratories (USA) supplied the microencapsulated phase change material. The core material inside the polymeric shell contains an n-alkane (n-C14, tetradecane) with a melting point (T_melt_) near 6 °C, which makes it suitable for low-temperature applications. The polymeric shell for encapsulating the tetradecane consists of melamine-formaldehyde. The mean average particle size and melting enthalpy of microencapsulated PCM are 0.021 mm and 195.5 J/g, respectively. More details about the actual size distribution can be found elsewhere^18^. The density of microencapsulated PCM (0.834 g/cm^3^) was measured by using Helium pycnometer (Micromeritics AccuPyc II, USA).

Asphalt binder 160/220 was used to prepare the PCM modified and reference asphalt mastics, and mixtures. Calcium carbonate (Nekafill 15) as a mineral filler supplied by KFN (Switzerland) was used to prepare the reference asphalt mastic. In the preparation of the mixtures, large and fine fractions of the mineral aggregates were type quartz supplied from Hard AG (Switzerland). Each aggregate size gradation was obtained from the quarry stockpile. In the laboratory, the aggregates were dried overnight in an oven at 105 °C followed by sieving using a sieve shaker.

For modified asphalt mastics, the blending of microencapsulated PCM with asphalt binder was conducted based on the percentage of mass addition (PCM/binder ratio = 0.5) by using a Speed Mixer model DAC 150.1 FVZ (Hauschild Engineering, Germany) at 2000 rpm over 2 minutes. The temperature of 120 °C at the time of blending was chosen to ensure proper blending of the asphalt binder with the microencapsulated PCM. The reference asphalt mastic test samples were prepared with calcium carbonate as mineral filler (density = 2.780 g/cm^3^) based on the equal volume replacement of PCM/binder ratio.

In asphalt mixtures, a mid-range-aggregate gradation type AC 8 S was used following the Swiss standard gradation specifications^30^ as shown in Table 1. In this study, the addition of approximately 9% of calcium carbonate (mineral filler) by weight of total dry aggregates, act as mineral filler in the conventional asphalt mixtures (reference). All the mixtures were prepared with 5.5% binder content by mass of the total mix. For the PCM modified asphalt mixtures, regarding the differences in densities, the substitution of the mineral filler with the microencapsulated PCM was carried out by the equivalent corresponding percentage of volume replacement (8.6%). The amount of PCM in asphalt mixtures is considered approximately the same as the amount used in the mastic study. Furthermore, in order to evaluate the influence of the incorporation method and its effect on the survival of the microcapsules as well as potential leakage of tetradecane^17,18^, 5 kg batches of asphalt mixtures were prepared following dry and wet processes.

**Table 1:** Mixture designs (type AC 8).

Sieve Size (mm)	Limits (%wt. passing)^30^	Reference (%wt. passing)	Reference (%wt. retained)	Reference (%vol.)	PCM (%wt. retained)	PCM (%vol.)
11.2	100	100	0	0.0	0.0	0.0
8	90–100	96	4	4.0	4.0	4.0
5.6	72–93	84	12	12.1	12	12.1
4	58–81	72	12	12.0	12	12.0
2	38–61	47	25	25.1	25	25.1
1	25–45	31	16	16.1	16	16.1
0.5	16–33	22	9	9.0	9	9.0
0.25	N/A	16	6	6.0	6	6.0
0.063	6–12	9	7	7.0	7	7.0
Filler	N/A	0	9	8.6	—	—
PCM	N/A	—	—	—	2.9	8.6

In the wet process, initially, the asphalt binder was heated to the blending temperature (140 °C) and was stirred to ensure uniform heat distribution before adding the required amount of microencapsulated PCM or of the mineral filler. Then, the speed mixer was used to blend the PCM or mineral filler with asphalt binder for 2 minutes at 2000 rpm. Finally, these mastics (PCM or reference mineral filler) were poured on the rest of 140 °C pre-heated mineral aggregates for the final mixing in a conventional mechanical laboratory mixer. For the dry process, the PCM (or mineral filler) were directly incorporated to the pre-heated aggregates (140 °C) and pre-mixed during 90 seconds prior to adding the hot asphalt binder and final mixing.

After mixing, the mixtures were left in the oven for two hours already set to the compaction temperature (130 °C). Gyratory compaction was used to compact two sets of test samples (Diameter 100 mm) from each asphalt mixture, which corresponded to an assumed 30 million equivalent single axle loads as specified by Superpave mix design. For that reason, the number of gyrations corresponding to initial compaction (N_initial_), design compaction (N_design_), and maximum compaction (N_max_) was determined as equal to 8, 100 and 125 gyrations, respectively^31^. During the compaction process, an angle of gyration 1.25° and 600 kPa vertical force were applied to the mixtures.

### Thermal characterization of asphalt mastic

As in previous works^18^, differential scanning calorimetry (DSC Mettler Toledo, USA) was used to analyse the mineral (reference) and PCM modified asphalt mastics. Test samples of 10 mg were prepared for the DSC analysis. Firstly, the samples were cooled to −40 °C and maintained at this temperature for 5 min. Then they were heated up to 100 °C and kept at this temperature for 5 min before cooling again to −40 °C where it remained for 5 min until finally being heated up to 100 °C. The cooling and heating ramps were conducted with a rate of 10 °C/min. Each formulation was analysed at least three times for reproducibility purposes and for standard deviation calculation.

### Rheological performance of asphalt mastic

In this study, a dynamic shear rheometer (DSR Physica MCR 301 DSR, Anton Paar GmbH., Austria) was used to analyse the rheological properties of the mineral (reference) and PCM modified asphalt mastics. As per EN 14770^32^, the parallel plate configuration with diameters of 8 mm corresponding to thicknesses of 2 mm was used. A low-temperature ramp from 20 °C to −10 °C (corresponds to a cooling rate of −0.44 °C/min) was used while applying oscillatory shear strain with constant strain amplitude (0.1%) and frequency (1 Hz) in order to avoid damaging the sample^18^. Peltier systems H-PTD200 and P-PTD200 (Anton Paar GmbH., Austria) were used for cooling.

### Thermal behaviour of modified asphalt mixtures

To evaluate the thermal effect of PCM on the temperature performance of asphalt mixtures, specimens were subjected to continuous cooling and heating cycles inside a climate chamber. Each thermal cycle consisted of an initial conditioning time of 150 min at 10 °C followed by a cooling ramp to −10 °C in 90 min (ca. −0.23 °C/min) and then, a conditioning of 150 min at −10 °C followed by heating ramp back to 10 °C in 90 min. This thermal cycle was repeated five times. In order to measure and record the thermal evolution, thermocouples were attached to the surface of different test samples. In addition to this, thermal images were captured using professional infra-red camera (Testo Ltd, United Kingdom) with full videometric resolution of 320 × 240 pixels.

### Mechanical response of modified asphalt mixtures

Using indirect tensile tests (IT-CY) on 100 mm diameter by 65 mm height cylindrical specimens, the European Standard EN 12697-26^33^ procedure was followed to characterize the so-called stiffness modulus of asphalt mixtures in the linear viscoelastic range (LVE) at low temperature. This was achieved by keeping applied strains under 50 μstr. A repeated load with a haversine waveform (max 10 kN, rise time 124 ms) with rest periods and pulse repetition of 3 s was applied along the vertical diameter of the specimen via the loading platens. The test samples were conditioned in a climate chamber at the test temperatures of 0 °C and 10 °C for 4 h before the tests in order to assure that the phase change took place. After the initial adjustment phase, the stiffness modulus was determined twice by calculating the average from the first and second five loading pulses using the ratio of force and the displacement as shown in Eq. (1). The reported modulus is the average of the two specimens.

1 $$E=\frac{F\cdot (\mu +0.27)}{z\cdot h}$$

where E is the calculated stiffness modulus [MPa], F is the peak load [N], z is the amplitude of the horizontal deformation [mm], *h* is specimen thickness [mm], μ is the Poisson’s ratio defined in the standard to be 0.35.
